# S11-2 Cooperative planning with coaching elements in childcare centres: a qualitative investigation of childcare directors' perspectives

**DOI:** 10.1093/eurpub/ckac093.056

**Published:** 2022-08-29

**Authors:** Christina Müller

**Affiliations:** Institut für angewandte Gesundheitswissenschaften, Coburg University of Applied Sciences and Arts, Coburg, Germany

**Keywords:** cooperative planning, early childhood education, care settings

## Abstract

**Background:**

Interventions to promote health-enhancing physical activity in early childhood education and care settings are most successful when tailored to the specific needs of each childcare centre and providing ongoing support to address context-specific barriers. Our research project therefore initiated organizational development processes in 12 childcare centres in Southern Germany supported by coaching and self-assessment tools. The staff of each centre was instructed to set three centre-specific SMART goals targeting physical activity and to use Goal Attainment Scaling in order to track the success at implementation. This qualitative study aims at exploring childcare centre directors' views on the guided planning process and identifying facilitators and barriers for its implementation.

**Methods:**

We conducted guided semi-structured interviews with the directors of the centres after the 12- month organizational development process. 9 out of 12 directors were interviewed. The interviews were recorded, transcribed and analysed using qualitative content analysis with inductive category development.

**Results:**

Childcare directors mainly considered themselves to be the person with the greatest responsibility for the success of the process. The coaching was regarded as helpful for structuring the process, involving the whole team and becoming clearer about goals. Several factors were identified as facilitators: a beneficial personnel situation, the intrinsic motivation of staff, good team cooperation, a high priority of physical activity, previous experience with similar projects, pressure for change, individual drivers, a good infrastructure, parents' support and support from the administrating organization. Reported barriers included team conflicts, lack of willingness to accept change and shortness of time.

**Conclusions:**

Several contextual and interpersonal factors seem to influence the extent to which a cooperative planning process can be implemented by the staff of a childcare centre. The results help understand the process of change as a complex interventional system in which the intervention cannot be considered separately from the context.

